# Adapting a Behavioral Weight Loss Intervention for Delivery via Facebook: A Pilot Series Among Low-Income Postpartum Women

**DOI:** 10.2196/formative.9597

**Published:** 2018-09-10

**Authors:** Valerie J Silfee, Andrea Lopez-Cepero, Stephenie C Lemon, Barbara Estabrook, Oanh Nguyen, Monica L Wang, Milagros C Rosal

**Affiliations:** 1 Division of Preventive and Behavioral Medicine Department of Medicine University of Massachusetts Medical School Worcester, MA United States; 2 Family Health Center of Worcester, Inc Worcester, MA United States; 3 Department of Community Health Sciences School of Public Health Boston University Boston, MA United States

**Keywords:** Facebook, health disparities, postpartum women, social media, weight loss

## Abstract

**Background:**

Efforts to translate evidence-based weight loss interventions, such as the Diabetes Prevention Program (DPP), to low-income postpartum women have resulted in poor intervention attendance and high attrition. Strategies that improve engagement and retention in this population are needed to maximize the reach of evidence-based weight loss interventions.

**Objective:**

The objective of this study was to adapt a DPP-based weight loss intervention (Fresh Start) for Facebook delivery and to evaluate its feasibility among low-income postpartum women.

**Methods:**

This study comprised 3 single-group pilot studies where feasibility outcomes iteratively informed changes from one pilot to the next. We paralleled the in-person program for Facebook delivery by translating the protocol to a content library of Facebook posts with additional posts from lifestyle coaches. Low-income postpartum women were recruited from Women, Infants, and Children (WIC) clinics in Worcester, Massachusetts. Participants were enrolled into a 16-week weight loss intervention delivered via Facebook. During the first 8 weeks, Facebook intervention posts were delivered 2 times per day, with additional posts from coaches aiming to stimulate interaction among participants or respond to participants’ questions and challenges. For the following 8 weeks, posts were delivered once per day without additional coaching. Feasibility outcomes were engagement (defined by number of likes, comments, and posts measured throughout intervention delivery), acceptability, and retention (survey at follow-up and assessment completion rate, respectively). Changes in weight were also assessed at baseline and follow-up.

**Results:**

Pilot 1 had a retention rate of 89% (24/27), and on average, 62% (17/27) of women actively engaged with the group each week during the 8-week coached phase. Mean weight loss was 2.6 (SD 8.64) pounds, and 79% (19/27) would recommend the program to a friend. Pilot 2 had a retention rate of 83% (20/24), and on average, 55% (13/24) of women actively engaged with the group weekly during the 8-week coached phase. Mean weight loss was 2.5 (SD 9.23) pounds, and 80% (16/24) would recommend the program to a friend. Pilot 3 had a retention rate of 88% (14/16), and on average, 67% (11/16) of women actively engaged with the group weekly during the 8-week coached phase. Mean weight loss was 7.0 (SD 11.6) pounds, and 100% (16/16) would recommend the program to a friend.

**Conclusions:**

Our findings demonstrated that a Facebook-delivered intervention was acceptable and could be feasibly delivered to low-income postpartum women. Future research is needed to evaluate the efficacy of a Facebook-delivered weight loss intervention.

## Introduction

Obesity rates are disproportionately high among racial and ethnic minority and low-income groups [1-3]. These disparities are even greater among women as 42% of those living below the poverty level are obese compared with 29% of women living above the poverty level [4]. Similarly, the prevalence of obesity is 54.8% in non-Hispanic black women and 50.6% in Hispanic women compared with 38% in non-Hispanic white women [5]. Pregnancy and postpartum weight retention places socioeconomically disadvantaged women at a higher risk for overweight and obesity as racial or ethnic minority and low-income women are more likely to exceed the Institute of Medicine guidelines for pregnancy weight gain and to retain weight after pregnancy [6-10]. As a result, excessive gestational weight gain and postpartum weight retention are risk factors for obesity over the life course [8], and thus, interventions to facilitate weight loss among low-income and racially or ethnically diverse postpartum women are needed.

A small body of literature has translated evidence-based interventions, such as the Look AHEAD (Action for Health in Diabetes) [11] and Diabetes Prevention Program (DPP) [12], to socioeconomically disadvantaged groups [13]. However, with a few exceptions [14], translating evidence-based protocols to real world settings has proven difficult. Previous behavioral weight loss trials designed for postpartum women have had limited impact and cited several challenges, including poor intervention attendance and high attrition rates due to difficulties finding transportation, securing childcare, and coordinating schedules [6,15-18]. Thus, innovative strategies that improve intervention engagement and overcome challenges of attendance and attrition among low-income postpartum women are needed.

Social media (eg, Facebook, Twitter, and Instagram) holds great promise as a potential means to deliver behavioral weight loss interventions, while overcoming previously identified challenges to participation and engagement among low-income postpartum women [19,20]. Social media usage is high among US adults, and Facebook is currently the most widely used social media platform, with 68% of adults currently using Facebook [21]. Facebook usage rates are also high among women and low-income groups. For example, 74% of female adults use Facebook, compared to only 62% of male adults. Social media use has penetrated even the very poor such that 66% of adults earning < US $30,000 per year use Facebook [21]. Individuals use social media to seek information on a variety of topics, including health and weight loss [19]. Users can also access social media at their convenience and overcome attendance barriers and burdens commonly experienced during in-person interventions (ie, transportation, childcare responsibilities, and scheduling) [14,19].

In multiple studies, social media has been one of the several components of behavioral interventions focusing on diet, physical activity, weight management, smoking cessation, and sun protection [20,22-26]. Recent literature reviews have concluded that weight loss interventions utilizing social media produce modest but statistically significant weight loss among overweight and obese individuals [20,27,28]. However, social media was mostly used in combination with other delivery modalities (eg, in-person groups, short message service (SMS) text messages) [6,29,30], and a few studies have evaluated the feasibility and effectiveness of delivering a weight loss intervention primarily via a publicly available social media network (eg, Twitter, Facebook) [20,22]. Furthermore, the existing evidence on social media-delivered interventions is based largely on white and high socioeconomic status samples, and additional research on the feasibility of interventions delivered via social media among socioeconomically disadvantaged populations is needed [6,28,31]. To examine the feasibility of social media as a delivery mode for behavioral weight loss interventions among low-income and minority postpartum women, this study translated a previously adapted, DPP-based weight loss intervention (The Fresh Start Trial) for low-income postpartum women to be delivered via Facebook. We describe a series of 3 pilot studies in which we evaluated the feasibility and acceptability of using Facebook as the primary intervention delivery modality. The iterative format of the pilot studies allowed us to test the Facebook-adapted intervention and refine the intervention materials and study methods based on feedback received after each pilot study. Methods, results, and lessons learned from each of the 3 pilot studies are described.

## Methods

### Design

All 3 pilot studies followed similar methodology unless otherwise noted. Each pilot study utilized a single-group pretest-posttest design. Study procedures were approved by the Institutional Review Board at the University of Massachusetts.

### Recruitment

Study participants were low-income postpartum women in Worcester, Massachusetts recruited from the Worcester Women, Infant, and Children (WIC) program over the course of 8 months [32]. Potentially eligible women were identified at their first postpartum appointment or via electronic records that identified likely eligible women based on their baby’s birthdate, body mass index, and language (ie, able to communicate in English). WIC providers prescreened women by completing a checklist of study pre-eligibility criteria based on chart information. Women who met these criteria were informed about the program during their next WIC visit. Pre-eligible women received a study fact sheet from the providers, who also asked the women about their interest in learning more about the study. Interested women provided their contact information. The study recruiter then contacted these women to explain the study further, ask additional eligibility questions, and determine interest in participating in the study. Eligibility criteria included the following: (1) childbirth in the previous 6 weeks to 6 months; (2) age≥18 years; (3) body mass index ≥27 kg/m^2^; (4) English-speaking; (5) approved by their health care provider to participate in a weight loss program; (6) daily access to the internet; and (7) regular Facebook use, defined as at least once per week. Exclusion criteria were as follows: (1) unable or unwilling to give informed consent; (2) pregnant or planning to become pregnant during the study period; (3) psychiatric illness that limits their ability to participate; (4) medications that cause weight change; (5) no access to a telephone; and (6) planning to move out of the area within the study period. Eligible women provided verbal consent for the study staff to contact their health care provider to seek approval for their participation in the study. Formal written informed consent for study participation was obtained during the baseline visit, prior to the completion of study assessments.

### Intervention Description

#### Adaptation Process

Intervention content was adapted from the original Fresh Start intervention, a weight loss treatment protocol based on content from the DPP [11], adapted for mothers with young children [14,33]. Briefly, the original Fresh Start protocol involved an 8-week group-based curriculum delivered by a WIC nutritionist. The intervention format included a narrative component, group discussions, print materials, and access to fitness facilities, followed by 9 monthly follow-up telephone calls.

This intervention was adapted for delivery via Facebook. First, the research team held regular meetings to review the protocol for the in-person Fresh Start Trial [33], which was adapted from the DPP curriculum [34]. The team identified key constructs and topics from the sessions and created a draft library of Facebook posts that were originally copied verbatim from the in-person protocol. In accordance with the Fresh Start protocol [33], posts emphasized one new topic each week (eg, tracking food and beverage intake, reading nutrition labels) and the use of behavioral strategies including self-monitoring and goal setting. The language was then simplified, and messages were shortened into simple terms and short sentences. The adaptation process for the Facebook intervention using goal setting as an example as well as a collage of posts can be found in Multimedia Appendix 1 and Multimedia Appendix 2. As research has shown that Facebook engagement is higher when a post has a photo or video [19,35], videos and pictures from the in-person protocol were included in posts where applicable and supplemented by additional photos, infographics, and videos extracted from Web-based sources with special attention to maintaining the original message. These were created using Microsoft Publisher and Windows Media Player. To promote interaction among participants in the Facebook group, all posts ended with an open-ended question regarding the topic of the post [19]. Facebook posts were then systematically ordered into a “feed” based on the order and progression of the original protocol and previous social media marketing research reporting an ideal frequency of 1-2 Facebook posts per day [19,35].

Finally, 8 research staff members with experience in weight loss intervention and social media approaches participated in a mock Facebook group where posts were delivered and pilot-tested. These individuals were asked to provide feedback on the wording of the posts, including the language, pictures, and videos, and whether the posts emphasized behavioral weight loss strategies and principles of motivational interviewing. Posts were revised and finalized for the first pilot based on feedback from the mock group.

#### Intervention Procedures

The intervention consisted of an 8-week intervention phase followed by an 8-week maintenance phase delivered via a secret, private Facebook group, preceded by a 90-minute in-person orientation session. Each of the 3 pilots held 2 orientation sessions (one in the morning and one in the evening) that women attended based on their needs, and sessions were required for participation in the study. The orientation allowed women to meet other women in their Facebook group, provided instruction on how to join the Facebook group, and informed women about the rules of the Facebook group.

Women were also introduced to the concepts of goal setting and taught how to track their diet, physical activity, and weight loss using a commercial mobile app (MyFitnessPal) or paper records. They were also provided a scale, pedometer, workbook, measuring cups, and a 1-year gym membership to YWCA Central Massachusetts at no cost to them.

Following the orientation, participation in the Facebook intervention commenced. During the first 8 weeks, Facebook intervention posts were delivered 2 times per day (8 am and 4 pm), 7 days per week, from study-created Facebook accounts (one from the intervention coach and one from the assistant coach) via the social media management platform Buffer [22]. The intervention coach was a postdoctoral fellow with experience and training in behavioral weight loss intervention delivery, and the assistant coach was a doctoral student. The coaching tasks included liking and commenting on the women’s posts or comments, encouraging discussion and sharing of strategies to deal with challenges to goal attainment or weight loss among the women, answering questions, and providing support. Coaches also provided group-based feedback based on women’s answers to intervention prompts. For the second 8-week period of the intervention (weeks 9-16), Facebook posts were delivered once per day without additional input from coaches.

### Outcome Measures

#### Measures Assessment

Outcome measures for each pilot study focused on feasibility outcomes including retention, engagement, and satisfaction. [36]. Weight change also was assessed. Participants completed the survey and anthropometric measures at baseline (preintervention assessment) and 16-week follow-up (postintervention assessment). Additional engagement data were obtained from Facebook, as described below. Participants who completed the postintervention assessment received a $50 gift card incentive.

#### Height and Weight

Height and weight were measured at baseline and after 16 weeks by trained research staff using a stadiometer and digital scale, respectively, with participants removing their shoes.

#### Engagement

As in previous studies [19,22,23], engagement was operationalized as participants’ behavior and interactions with the Facebook group, including all likes, comments, and posts in each week. Facebook data were downloaded weekly using Facebook Downloader V5.0.1, and it included the number of comments, likes, and original posts from each participant over the course of the intervention. Additional survey questions asked women to self-report their level of engagement with a series of items that inquired how often they read the entire intervention posts and how often they read part of the intervention posts. Response options were on a 6-point scale ranging from “never (0% of the time)” to “almost always or always (90%-100% of the time).” Indiscernible Facebook engagement, or “lurking,” was also defined via the extent to which women read the intervention posts without commenting on it or liking it [37]. Specifically, women reported how often they read the entire post and did not respond by liking it or commenting on it on a 5-point scale ranging from “never” to “always.”

#### Acceptability

Survey items at the follow-up assessment asked about satisfaction with the intervention overall and satisfaction with the amount of weight lost during the program. Responses were rated on a 5-point scale ranging from “very dissatisfied” to “very satisfied.” Women also rated how helpful they found the program in helping them lose weight on a 5-point scale from “very unhelpful” to “very helpful.” We asked the participants how likely they were to participate in a similar weight loss program and how likely they were to recommend the program to a friend on a 5-point scale from “very unlikely” to “very likely.” Women also rated how often they felt supported by other participants in the group on a 5-point scale ranging from “never” to “always.” Finally, women rated the extent to which they felt the other participants were motivating and the extent to which they felt the coaches were helpful and motivating based on a 5-point scale from “strongly disagree” to “strongly agree.”

### Qualitative Group Discussion

Upon completion of the postintervention assessment, participants were invited to participate in a 60-minute group discussion, during which they provided feedback on their experience in the intervention*.* These discussions were led by an experienced facilitator, and a note taker was present. The discussions were recorded, and 2 members of the study team independently listened to the recordings, looking for concrete suggestions from the women to improve the Facebook intervention. The interventionists and facilitator then met and used an expert consensus approach to reach an agreement on what changes and modifications would be made to the intervention from pilot to pilot.

## Results

### Pilot 1 Results

#### Overview

A total of 29 women were initially enrolled in Pilot 1. However, 2 participants became ineligible during the intervention, one due to medical reasons and the other due to intake of a weight-altering medication (study exclusion criterion). Thus, the final sample for Pilot 1 was 27 women (Table 1). The retention rate for this pilot was 89% (24/27).

**Table 1:** Baseline characteristics of women participating in the three pilot studies.

Characteristics		Pilot 1 (n=27)	Pilot 2 (n=24)	Pilot 3 (n=16)
Age, mean (SD)		32.1 (5.6)	29.4 (6)	29.4 (4)
Body mass index, mean (SD)		35.1 (5.5)	38.2 (6)	34.9 (7)
**Race or ethnicity, n (%)**				
	Hispanic or Latina	11 (41)	5 (21)	7 (44)
	Non-Hispanic black	5 (19)	7 (29)	2 (13)
	Non-Hispanic white	8 (30)	9 (38)	6 (38)
	Asian	2 (7)	1 (4)	0 (0)
	Other	1 (4)	2 (8)	1 (6)
**Education, n (%)**				
	High school degree or less	13 (48)	7 (29)	4 (25)
	Some college or 2-year degree	8 (30)	10 (41)	9 (56)
	College degree or more	6 (22)	7 (29)	3 (19)
**Marital status, n (%)**				
	Single	10 (37)	8 (33)	7 (44)
	Married or living with partner	16 (59)	14 (53)	8 (50)
	Divorced or separated	1 (4)	1 (4)	1 (6)
**Facebook activity prior to enrollment, n (%)**				
	Posted a Facebook status once per week or more	17 (63)	18 (75)	11 (69)
	Posted a video or photo to Facebook once per week or more	16 (59)	16 (67)	10 (63)
	Commented on a friend’s Facebook post once per week or more	22 (82)	22 (92)	13 (81)

#### Engagement

Table 2 displays the engagement for all 3 pilots during the coached (weeks 1-8) and noncoached (weeks 9-16) phases of the intervention. On average, about 63% (17/27) women engaged in the group each week. Of the 24 women who completed the 16-week assessment, 71% (17/24) reported reading the entire intervention posts either most of the time or always and 42% (10/24) said that they read only part of the posts either most of the time or always. When asked about lurking, 1 woman reported never reading a post without commenting on it or liking it; 38% (9/24) women reported occasionally reading a post without commenting on it or liking it; 17% (4/24) women reported lurking half the time, 21% (5/24) women much of the time, and 21% (5/24) women always.

#### Weight Loss

Weight loss outcomes at the 16-week follow-up are presented in Table 3. Women lost an average of 2.6 (SD 8.64; range −23.4 to 14.4) pounds or 1.4% of their baseline weight (SD 4.4; range −12.4 to 5.8). At the 16-week follow-up, 63% (15/24) women lost weight and 38% (9/24) women gained weight.

#### Acceptability

A majority (75%, 18/24) of the participants reported being satisfied or very satisfied with the intervention, and 19 out of 24 women (79.2%) felt the program was somewhat or very helpful in facilitating their weight loss (Table 3). Furthermore, 63% (15/24) women felt supported by other participants in the group at least half of the time, and 67% (16/24) felt that the other women in the group were motivating. Finally, all 24 women (100%) felt that the coaches were helpful and motivating.

#### Lessons Learned and Intervention Adaptations

We learned several key lessons from Pilot 1 based on the group discussions and Facebook group engagement data. First, we explored intervention posts with lower engagement, as defined by <5 comments on the post or <3 women who commented on the post, to identify key themes or similarities between posts with poor engagement. From our review of engagement data, we discerned that goal setting posts, posts with lengthy videos, and posts asking multiple questions were associated with lower engagement among the participants. As a result, we edited goal posts to include sample goals, exchanged long videos for infographics, and reduced the word count of lengthier posts to simplify the posts further. Of note, when iterations were made to the approach to behavioral strategies, the core evidence-based behavioral strategies were maintained across groups. During the group discussions, we learned that women were more likely to read and comment on a post that included helpful pictures and visuals. For example, a few of the women shared that they took “screenshots” of the intervention pictures and saved them to the photo library on their mobile phones to be able to access them later. We therefore carefully reviewed intervention posts that included photos (eg, a picture of a mom walking) versus infographics (eg, a diagram of healthy snacks with calorie amounts), and where applicable, we enhanced posts to include infographics.

Second, during the first few weeks of the intervention, women reported via Facebook posts to the group and private messages to the coaches that they were having difficulties tracking their diet or activity using the recommended app (ie, MyFitnessPal). We thus modified intervention posts during the first 2 weeks to include posts that provided support in learning to use the app. Specifically, we created 4 new Facebook posts for Pilot 2 that included both videos and infographics and directed women on key features of tracking their food using the app.

Finally, during the group discussions, women reported that they wished there were more opportunities for the participants to meet in person and suggested that this be accomplished via increased utilization of the free YWCA membership. Based upon this suggestion that in-person interactions be in the form of workout groups or other exercise participation, during the coached phase of Pilot 2, participants received Facebook posts with invitations to join and participate in an exercise class at the YWCA with other women in the group and one of the coaches. Class type (eg, Zumba, yoga) and day and time varied each week to facilitate the attendance of women with different scheduling needs.

**Table 2:** Measures of intervention engagement for the three pilot studies.

Measures		Pilot 1 (n=27)		Pilot 2 (n=24)		Pilot 3 (n=16)	
		Coached phase	Noncoached phase	Coached phase	Noncoached phase	Coached phase	Noncoached phase
**Engagement measures per participant, mean/week (SD)^a^**							
	Original posts	2 (2.5)	0 (0.62)	3 (3.8)	0 (0.3)	3 (2.6)	0 (0.3)
	Comments	18 (25.2)	2 (4.4)	24 (31.2)	4 (6.6)	16 (15.5)	3 (4.9)
	Likes	33 (57.7)	5 (8.8)	19 (27.1)	3 (4.5)	19 (21.8)	2 (2.8)
**Women engaged, n (%)**							
	Women who engaged in all 8 weeks	8 (33)	1(4)	4 (20)	2 (83)	4 (29)	1(19)
	Women who engaged in ≥3 out of 8 weeks	21 (88)	11 (41)	17 (85)	11 (46)	12 (87)	6 (38)
	Women who did not engage	1 (4)	10 (37)	3 (13)	9 (38)	0 (0)	6 (38)

^a^Engagement indicators are averages rounded to the nearest whole number.

**Table 3:** Weight change and participant satisfaction outcomes in the three pilot studies.

Outcomes		Pilot 1 (n=24)	Pilot 2 (n=20)	Pilot 3 (n=14)
**Participant weight loss outcomes**				
	16-week weight change (lbs), mean (SD)	−2.6 (8.64)	−2.5 (9.23)	−7.0 (11.6)
	16-week weight change (%), mean (SD)	−1.4 (4.38)	−1.2 (3.97)	−3.6 (5.6)
	16-week body mass index change, mean (SD)	−0.5 (1.5)	−0.5 (1.6)	−1.3 (2.2)
	Lost any weight at 16 weeks, n (%)	15 (63)	10 (50)	10 (71)
	Gained any weight at 16 weeks, n (%)	9 (38)	9 (45)	2 (14)
**Participant satisfaction outcomes**				
	Satisfied with the program, n (%)	18 (75)	16 (80)	9 (64)
	Program helpful in facilitating weight loss, n (%)	19 (79)	12 (60)	11 (79)
	Would recommend program to a friend, n (%)	19 (79)	16 (80)	14 (100)
	Would continue program after study ends, n (%)	15 (63)	18 (90)	13 (93)

### Pilot 2 Results

#### Overview

Pilot 2 had initially enrolled 25 participants. However, 1 woman became ineligible due to pregnancy, leading to a sample of 24 women (Table 1). The retention rate for this pilot was 83% (20/24).

#### Engagement

An average of 13 (55.2%) women engaged in the group each week. Of the 20 women who completed the 16-week assessment, 70% (14/20) reported reading the entire intervention post either most of the time or always, and 65% (13/20) women said that they read only part of the post most of the time or always. When asked about lurking behavior, 35% (7/20) women reported occasionally reading a post without commenting on it or liking it; 25% (5/20) women reported lurking half the time, 25% (5/20) women much of the time, and 5% (120) woman always.

#### Weight Loss

Women lost an average of 2.5 (SD 9.23; range −26.2 to 8.8) pounds or 1.2% of their baseline weight (SD 3.97; range −9.8 to 3.8; Table 3). Half (10/20, 50%) of the women lost weight, 5% (1/20) woman maintained her baseline weight, and 45% (9/20) women gained weight.

#### Acceptability

A majority (16/20, 80%) of the women reported being satisfied or very satisfied with the intervention, and 60% (12/20) women felt the program was somewhat or very helpful in their weight loss effort. Furthermore, 65% (13/20) women felt supported by other women in the group at least half of the time, and 60% (12/20) women felt the other women in the group were motivating. Finally, 90% (18/20) women felt that the coaches were helpful, and 95% (19/20) women felt that the coaches were motivating.

#### Lessons Learned and Intervention Adaptations

Observations and findings from Pilot 2 provided additional insight on how to enhance the intervention. First, women endorsed opportunities to attend group exercise classes with one of the intervention coaches at the YWCA. However, only 3 of the women ever attended these classes. Challenges to attendance included lack of time, class timings not working well with their schedules, and childcare responsibilities (babysitting). Second, women expressed that they wished more women would have engaged actively in the Facebook group. Factors that got in the way of active engagement included feeling uncomfortable posting about themselves and not wanting to post if they were not experiencing successful weight loss. In terms of intervention content, women expressed that they were less likely to read posts that were too long and endorsed posts that elicited responses via questions and included visuals. Examples of helpful visuals included pictures of appropriate portion sizes, recipe substitution ideas, and number of calories of specific commonly consumed foods.

From the lessons learned in Pilot 2, we made several modifications for Pilot 3. First, we again carefully reviewed the posts based on the women’s feedback, as well as posts that elicited low engagement (<5 comments or <3 women commenting). We adjusted the language to make them more concise and engaging, and where applicable, we replaced some of the text with a picture or video. In response to feedback regarding the use of gym, we extended the orientation by 5 minutes to include more information about the YWCA and provide women with a map to the facility and the group exercise schedule. We were also able to secure babysitting at the YWCA for 2 hours, 3 days per week: 1 day in the morning, 1 day in early afternoon, and 1 day in the late afternoon or early evening. To encourage women to post more in the group, we added 2 posts in the first week of the intervention; one asked women to introduce themselves to the group and one encouraged women to post in the group regardless of their motivation throughout the program (eg, when they lost weight vs when they did not lose weight; when they had a good week vs when they had a bad week).

### Pilot 3 Results

#### Overview

Pilot 3 had 17 enrolled participants. However, 1 woman became ineligible during the study due to a new pregnancy, leading to a final sample of 16 women (Table 1). The retention rate in Pilot 3 was 88% (14/16).

#### Engagement

On average, 67% (11/16) women engaged in the group each week. Out of the 14 women who completed the 16-week assessment, 43% (6/14) women reported reading the entire intervention post most of the time or always (≥75% of the time), and 43% (6/14) said that they read part of the post most of the time or always. When asked about lurking behavior, 36% (5/14) women reported occasionally reading a post without commenting on it or liking it; 21% (3/14) women reported lurking half the time, 36% (5/14) women much of the time, and 7% (1/14) woman always.

#### Weight Loss

During Pilot 3, women lost an average of 7.0 (SD 11.6; range −31.8 to 13.8) pounds or 3.6% of their baseline weight (SD 5.62, range −11.9 to 6.1; Table 3). Out of 14 women, 71% (10/14) women lost weight, 14% (2/14) women maintained their baseline weight, and 14% (2/14) women gained weight.

#### Acceptability

A majority (9/14, 64.3%) of the women reported being satisfied or very satisfied with the intervention, and 79% (11/14) women felt the program was somewhat or very helpful for their weight loss effort. Furthermore, 64% (9/14) women felt supported by other women in the group at least half of the time, and 57% (8/14) women felt the other women in the group were motivating. Finally, all 14 (100%) women felt that the coaches were helpful and 93% (13/14) women felt that the coaches were motivating.

#### Lessons Learned

We learned several key lessons from the women in Pilot 3. Women reported that they would have liked there to be more interaction among the women in the group, and a few suggested more opportunities for in-person meetings or get-togethers (eg, Pilates in the park, walking around the neighborhood). However, like Pilot 2, a discrepancy existed between these suggestions and attendance in in-person group opportunities, as none of the women in Pilot 3 utilized the babysitting or attended the classes at the YWCA with one of the coaches. Women also reported several factors that contributed to their engagement with the Facebook group, including the time of day of the post, how much time had passed since the post, and how many other women had commented on the post. Women also offered several suggestions for improving communication between each other within the Facebook group, such as exchanging phone numbers during the orientation session, having nonweight loss related ice breakers on the Facebook group (eg, asking about work schedules or how many kids women have), and having weekly step or weight loss competitions. Finally, women provided feedback on intervention content and reported that they found practical tips related to meal planning, proper food storage, food preparation, and recipes to be particularly helpful.

Based on the findings and feedback from Pilot 3, as well as Pilots 1 and 2, important lessons that could inform further intervention refinement were the intervention posts that included concise language; open-ended questions that elicited responses; infographics and content related to weight loss progress, calorie goals and budgeting; and practical tips for meal planning and cooking. Furthermore, while women in all 3 pilots liked receiving the intervention content via Facebook, they desired more social support from one another and suggested solutions, such as a longer orientation session, more ice breakers and discussions on Facebook, and face-to-face interactions to facilitate this support. It is important to note, however, that when women in these pilots were offered more opportunities for in-person interaction, they did not utilize them.

## Discussion

### Principal Findings

This series of 3 pilot studies demonstrated that an adapted behavioral weight loss intervention delivered primarily via Facebook is feasible and acceptable among low-income postpartum women. On average, more than half of the women in each of the 3 pilots actively engaged (measured by likes, comments, and posts) in the group each week during the coached phase of the intervention (weeks 1-8). Furthermore, women were highly receptive as shown by the fact that most of them found that the intervention was helpful for losing weight, the coaches were supportive, and they would be likely to recommend the program to a friend. Given this high level of receptivity, the finding that satisfaction decreased slightly from pilot 1 to pilot 3 is difficult to interpret. Finally, women in Pilot 3 lost an average of 7 pounds compared with those in Pilots 1 and 2 who lost 2.6 pounds and 2.5 pounds, respectively.

Several previous studies among postpartum women enrolled in WIC programs have observed no significant changes in body weight following a variety of intervention delivery modalities including digital virtual discs (DVDs) and support group teleconferences [16], peer-led support groups [15], and mobile apps [38]. Our pilot series suggests that intervening via Facebook as a primary delivery modality is feasible and may be acceptable in this population. Additionally, although our findings are preliminary, they suggest that the intervention may have potential to surpass weight loss outcomes observed with other intervention modalities targeting WIC clients. However, these 3 pilots were conducted without a control group, and confounding factors (eg, time of year, weather) may have contributed to the improved weight loss across pilots. For example, the first study took place during the holiday season (October-January), the second study right after the new year and the third study during the summer. Thus, a larger randomized controlled trial to evaluate the effect of a Facebook-delivered weight loss intervention on short- and long-term weight loss among low-income postpartum women is warranted.

While direct comparisons with in-person interventions are not possible due to different delivery modalities, our results suggest slightly higher engagement levels. For example, in the original Fresh Start pilot intervention, 26% (7/27) of the women attended the first 3 weekly sessions, while 54% (36/67) of women across our 3 pilots participated in the Facebook group at least once in each of the first 3 weeks [14]. Another study among WIC women in West Virginia (N=151) reported that 47.4% (72/151) of the women attended at least 1 session, and 57% (86/151) never attended a group session [15]. Comparatively, 94% (62/67) of women across all 3 pilots actively engaged at least once during the 8-week coached intervention. Finally, the mean number of sessions attended in the aforementioned study was 3.6 out of 10 [15], which was similar to the mean 3.8 (out of 8) in in-person group sessions attended by women in the Active Mother Postpartum trial (N=450) [17]. Women across all 3 pilot studies engaged, on average, in 5 out of 8 weeks during the coached phase.

Despite a higher participation in the Facebook-delivered intervention, active engagement decreased throughout the intervention, particularly after the removal of the coach, in all 3 pilots. Instead, we observed an increase in passive engagement given that with fewer women liking, posting, and commenting, more women reported lurking. Previous research with low-income postpartum women has also consistently demonstrated poor engagement in weight loss intervention sessions [15,16,39]. These findings also are consistent with previous social media-delivered interventions where engagement declined over the course of the intervention [28].

To improve active engagement across the 3 pilots, we included additional opportunities for in-person exercise classes at the YWCA based on the women’s suggestions. However, when provided the opportunities to meet in person, women did not participate. This is an important factor for researchers to consider when evaluating available resources for interventions, and additional research is required to better understand this discrepancy. Future research may consider utilizing user experience design, which observes individuals in their daily lives, to better inform intervention design{Yardley, 2015 #1414}. This methodology may serve to close the gap between participant suggestions or desires and actual behaviors.

One approach to sustain engagement could be to enhance the coaching component of the intervention. As stated above, women across the 3 pilot studies were very receptive to the coaching aspect of the intervention, and active engagement declined considerably from the coached to noncoached phase of the study. Furthermore, while previous studies adapting the DPP have provided personalized feedback regarding weight, diet, and physical activity changes, feedback in this study was limited as it was based on women’s responses to posts rather than a thorough review of individual self-monitoring logs. Future studies may enhance the coaching component by providing more personalized feedback in addition to group-based feedback. Another approach could be to intervene with dyads or cohorts of individuals who already know each other [29,40,41]. For example, a pilot study evaluating the Mums Step it Up Facebook app used a snowballing recruitment method in which women enrolled in phase one recruited 3-7 of their friends to join their team in phase 2 in order to enhance the social nature of the intervention [40]. A third approach has been to incentivize participants based on the number of posts they contribute to the Facebook group [42]. Finally, some recent studies have investigated the type of intervention content that stimulates the highest level of engagement [24,43-46]. One study found that polls (ie, posts asking participants to suggest a tip for others) and weight-related posts, compared with recipe-related posts, nutrition information, and news, were the most engaging among participants in a 4-month weight loss study [43]. Another study found that polls and photos were the most engaging posts in a weight loss study among college students [45]. Together, these strategies, among others, may be critical to exploring how we can optimize the short- and long-term engagement of postpartum women in a Facebook-delivered intervention.

### Limitations

This study has several limitations. First, the sample sizes of the 3 pilots were relatively small and decreased over the 3 pilots due to declining WIC enrollment during our study period (October 2016-August 2017) and study time constraints. A limitation in our measures was that we were not able to obtain objective measures of lurking behavior or directly relate changes in the types and content of posts to engagement. This suggests that women may have had greater exposure to the intervention that was not reflected in our measures of engagement, and future studies that utilize social media to deliver behavioral weight loss interventions should investigate strategies or intervention iterations specifically related to engagement (eg, types of posts, post content) to sustain participation over time. Additionally, we were unable to conduct mediation analysis to determine the influence of engagement as a mediator of weight loss given that the sample size of our pilot studies was small. This study recruited women who were regular Facebook users, which may limit the generalizability of our findings. However, we found that access to social media was not a major barrier to women participating in this study, and only 2 women were ineligible due to limited Facebook use. This is consistent with recent research suggesting that low-income and minority populations have similar technology access to other population subgroups. As of 2017, internet usage, smartphone ownership, and social media usage were similar among Hispanic (88%, 75%, and 74%), and black (85%, 72%, and 63%) adults compared with white adults (88%, 77%, and 69%) [47,48]. While all 3 pilot studies sought qualitative feedback from participants, we did not conduct a formal in-depth qualitative analysis of the group discussions. Future studies may consider a mixed-methods approach to further understand the opportunities and challenges of this intervention modality. Lastly, an important challenge of delivering interventions via publicly available social networks is the ever-changing settings, interfaces, and features. For example, between Pilot 1 and Pilot 3 of this study, Facebook added reactions, an extension of likes where users could click “love,” “haha,” “wow,” “sad,” or “angry.” Because the existence of this feature was not consistent across pilots, we were not able to investigate engagement based on reaction types. While changes in the features of social media platforms are often difficult to predict, future investigators should consider the potential for network updates and the extent to which unforeseen changes may impact the intervention delivery and potentially its outcomes.

### Conclusions

Social media-delivered behavioral weight loss interventions show great promise due to their high potential of reaching low-income diverse individuals, reducing intervention burden, and decreasing the cost of weight loss intervention delivery. The findings from this series of 3 pilot studies demonstrated that a Facebook-delivered intervention was acceptable and could be feasibly delivered to reach low-income postpartum women. Future research is needed to evaluate the efficacy and sustainability of delivering a weight loss intervention via Facebook.

## Appendix

Multimedia Appendix 1 Step-by-step depiction of the adaptation process for Facebook posts.

Multimedia Appendix 2 Collage of Facebook posts.

## Abbreviations

DPP: Diabetes Prevention Program
WIC: Women, Infants, and Children
