# Supplementary figures and images for: Adapting a Behavioral Weight Loss Intervention for Delivery via Facebook: A Pilot Series Among Low-Income Postpartum Women
Source: JMIR Form Res. 2018 Sep 10;2(2):e18. doi: 10.2196/formative.9597 (PMC6334676; doi:10.2196/formative.9597)

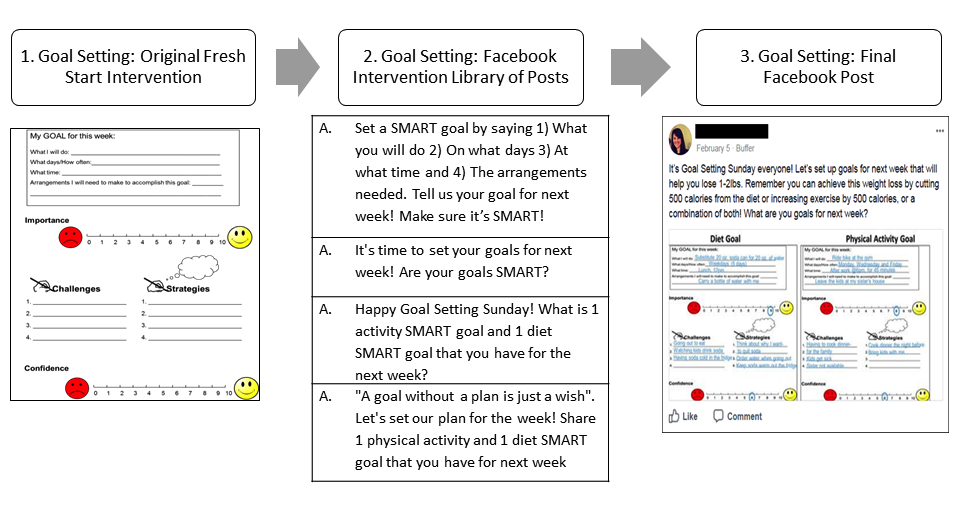

Supplement: Multimedia Appendix 1 [file formative_v2i2e18_app1.png]

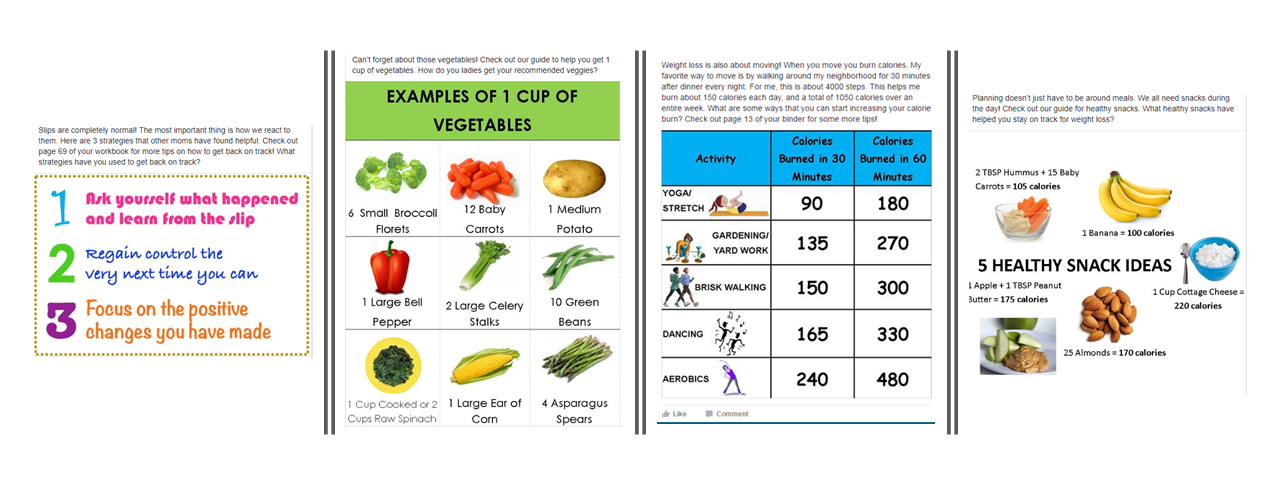

Supplement: Multimedia Appendix 2 [file formative_v2i2e18_app2.png]
